# Hypoxic Exercise Exacerbates Hypoxemia and Acute Mountain Sickness in Obesity: A Case Analysis

**DOI:** 10.3390/ijerph18179078

**Published:** 2021-08-28

**Authors:** Jing Xu, Jinshu Zeng, Yelei Yan, Fei Xu

**Affiliations:** School of Physical Education, Hangzhou Normal University, Hangzhou 311121, China; 2020112005013@stu.hznu.edu.cn (J.X.); jinshuzeng102@gmail.com (J.Z.); 2019112005028@stu.hznu.edu.cn (Y.Y.)

**Keywords:** obesity, acute mountain sickness, hypoxemia, hypoxic therapy, hypoxic exercise, hypoxic rest

## Abstract

Acute mountain sickness (AMS) is a common syndrome characterized by headache, dizziness, loss of appetite, weakness, and nausea. As a major public health issue, obesity has increased in high altitude urban residents and intermittent commuters to high altitudes. The present study investigated acute hypoxic exposure and hypoxic exercise on hypoxemia severity and AMS symptoms in a physically active obese man. In this case analysis, peripheral oxygen saturation (SpO_2_) was used to evaluate hypoxemia, heart rate (HR) and blood pressure (BP) were used to reflect the function of autonomic nervous system (ANS), and Lake Louise scoring (LLS) was used to assess AMS. The results showed that acute hypoxic exposure led to severe hypoxemia (SpO_2_ = 72%) and tachycardia (HRrest = 97 bpm), and acute hypoxic exercise exacerbated severe hypoxemia (SpO_2_ = 59%) and ANS dysfunction (HRpeak = 167 bpm, SBP/DBP = 210/97 mmHg). At the end of the 6-h acute hypoxic exposure, the case developed severe AMS (LLS = 10) symptoms of headache, gastrointestinal distress, cyanosis, vomiting, poor appetite, and fatigue. The findings of the case study suggest that high physical activity level appears did not show a reliable protective effect against severe hypoxemia, ANS dysfunction, and severe AMS symptoms in acute hypoxia exposure and hypoxia exercise.

## 1. Introduction

In some individuals, hypoxia occurs when ascending to high altitudes due to the decrease in barometric pressure [1]. Generally, people or animals rising from a plain to a high altitude of more than 2500 m will develop high altitude disease including various clinical syndromes within 6 to 12 h [2]. The high-altitude disease varies depending on altitude and exposure type. Acute mountain sickness (AMS) is a common syndrome characterized by headache, dizziness, loss of appetite, weakness, and nausea. The incidence of AMS is 9% to 75% [2,3], and the risk factors are related to the rate of ascent, altitude reached and individual susceptibility, such as age, ethnicity, exercise habits and obesity [4,5,6]. In a study of obese participants, Ge et al. [7] showed that obese patients’ incidence of AMS is 78% during the period of hypoxia night sleep.

As a public health issue, obesity has become a major health burden. According to the estimates recently issued by the World Health Organization, the total number of obese and overweight adults (age ≥ 18 years) was about 600 million and more than 1.9 billion in 2014 [8]. In recent years, hypoxic therapy (prevention and treatment of obesity through hypoxic exposure and/or hypoxic exercise) as a novel and effective tool has been widely used for the treatment of obesity [9,10,11]. A series of studies have confirmed that people long residing in high altitude have a significantly lower obesity-related disease and body fat percentage (fat %) [12,13]. In view of obesity have a variety of effects on physiological characteristics of individuals, especially on the respiratory system, such as respiratory muscle inefficiency and a heightened demand for ventilation [14]. However, hypoxia therapy is a double-edged sword. Owing to obesity’s frequent comorbidities, the negative effects of hypoxia therapy probably increase health risk factors due to susceptibility of AMS.

Although the specific physiological mechanisms of AMS are still unclear, Roach et al. [15] confirmed that altitude hypoxia is the underlying cause of AMS. Hypoxia is classified as acute, chronic, and intermittent hypoxia according to the duration of exposure to high altitude. In most cases, AMS patients exhibit abnormal physiological characteristics including low ventilation and excessive desaturation [16]. Currently, obesity has increased in high-altitude urban residents [17], as well as the intermittent commuters to high altitudes [18]. Meanwhile, an increasing number of studies indicate that obese people are more sensitive to developing AMS under hypoxic rest [7,19,20]. As evidenced by a series of studies found that the severity of hypoxemia is a promising tool for predicting AMS under acute hypoxic rest [16,21,22]. But the results are inconsistent [23]. Considering that people in the highlands have not always been at rest, and hypoxic therapy usually uses hypoxic exposure and/or hypoxic exercise to prevent and treat obesity [9,10,11]. Researchers believe that monitoring hypoxemia during hypoxic exercise could more effectively predict AMS [5]. Specifically, a previous study showed that hypoxic exercise exacerbates hypoxemia while aggravating the incidence and severity of AMS in physically active males [5].To some extent, actually, there are similarities between high altitude residence (trekking, skiing, and other basic physical activity, etc.) and hypoxia therapy. However, to the best of our knowledge, limited studies observed the combined effect of hypoxia and exercise on the developing of hypoxemia and AMS in obese people. Therefore, the aim of our study was to investigate the acute hypoxic exposure and hypoxic exercise on hypoxemia severity and AMS symptoms in a physically active young man. We hypothesized that hypoxic exercise exacerbates hypoxemia and induces acute mountain sickness in an obese patient, even if this case is physically vigorous.

## 2. Materials and Methods

### 2.1. Participants

Participants were publicly recruited via posting advertisement, telephone, bulletin boards, and student society organization outreach on the campus. Male college students were included after screening by the international physical activity questionnaire short-form (IPAQ-SF). The other inclusion criteria were that: (1) met basic criteria for being physically active [24]; (2) lived in the plains (altitude < 800 m); (3) not had been exposed to high altitude hypoxia (altitude ≥ 1500 m) before 6 months prior to the experiment; (4) non-smoking and no history of neurological or cardiovascular disease; (5) and not intake stimulants or any other drugs. After verification screening and inclusion criteria, 23 subjects from 86 volunteers were recruited for the present study. All participants signed the informed consent form before the formal experiment. This study complied with the Declaration of Helsinki and was approved by the Institutional Review Board of Hangzhou Normal University (IR10346-SPT-2020). Participants could withdraw at any stage from the experiment according to their physical condition, feeling, and willingness.

Among the included 23 physically active participants, we found an anomaly 22-year-old male student, who is a collegiate football player. Although he meeting our inclusion criteria, his body mass index (BMI) and body composition test results revealed that he was an obese patient. Therefore, here we report his individual detailed data as a case analysis.

### 2.2. Normoxic Base Values Testing and Familiarization

Participants were measured for normoxic base values before 1 week prior to hypoxic exposure. They were asked to avoid intensive exercise and prohibited from ingesting stimulating substances such as coffee and alcohol for 48 h prior to the experiment. The normoxic base values were measured in a chamber with a temperature of 20–24 °C, relative humidity of 46–67%, and a barometric pressure of 1014–1021 hPa. All participants were instructed to wear slim-fit and light-weight sportswear during the familiarization period and throughout the experimental procedures, weight and body composition were measured on barefoot by bioelectric impedance analysis (Inbody 720, Biospace Co., Seoul, Korea). A constant load exercise at 80 watts and 60 revolutions per minute (rpm) was performed on a supine bicycle, and the peripheral oxygen saturation (SpO_2_), heart rate (HR) and blood pressure (BP) were recorded during the test. In the familiarization period, participants should be familiar with the test instrument and procedures at least twice (any accidents during the familiarization period will not be counted).

### 2.3. Acute Hypoxic Exposure for 6 h

**Figure 1:**
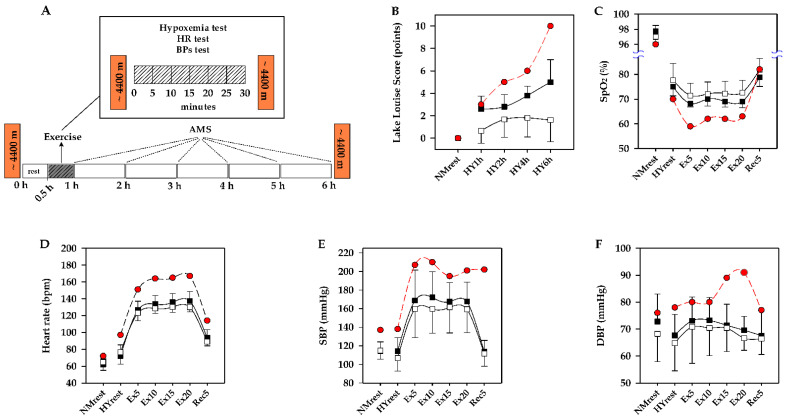
Protocol and changes of hypoxemia, HR and BP during acute hypoxic exposure and hypoxic exercise. Note: (**A**) protocol of hypoxia exposure 6 hours, (**B**) changes of Lake Louise score, (**C**) changes of SpO_2_, (**D**) changes of heart rate, (**E**) changes of SBP, (**F**) changes of DBP. One obese patient (solid circle), AMS (open square), nonAMS (solid square). NMrest, the rest of normoxia; HY, hypoxia; HYrest, the rest of hypoxia; Ex, exercise; Rec, recovery. AMS, acute mountain sickness (LLS ≥ 3 points); nonAMS, absence of AMS (LLS < 3 points); SpO_2_, peripheral oxygen saturation; SBP, systolic blood pressure; DBP, diastolic blood pressure; The datum of AMS and nonAMS group have not been published yet.

Participants were exposed to a normobaric hypoxia chamber (simulated~4400 m, FiO_2_: 11.7% to 11.2%) for 6 h (30-min hypoxic rest, 30-min hypoxic exercise and 5-h hypoxic rest, see Figure 1A). After the participants entering the hypoxia chamber, the SpO_2_, HR, BP were continuously measured during the 30-min hypoxic rest and 30-min constant-load hypoxic exercise period. Subsequently, participants were sustained hypoxic exposure for the remaining 5 h in the hypoxia chamber. During the 5 h, the participants were allowed to eat a meal, rest up-right or semirecumbent, and nap when they felt sleepy.

### 2.4. Constant Load Exercise Tests

After a 30-min resting period of acute hypoxic exposure, participants performed a 30-min exercise constant load exercise test including 5-min rest, 20-min constant load exercise (80 W, 60 rpm) in supine position (GE Ergoselect 1000LP vehicle-mounted test hardware, GE Healthcare, New York, NY, USA) and 5-min recovery (Figure 1A). SpO_2_ (Nonin 3100 wristox pulse oximeter, Concord Health Supply, Chicago, USA), HR (Polar RS800 Electro Öy, Kempele, Finland) and BP (GE Ergoselect 1000LP vehicle-mounted test hardware, CE Healthcare, New York, NY, USA) were recorded every 5 min during the hypoxic exercise period. The ECG (Cardiocollect Hand-held 12-lead, HCE Medical, Ipswich, UK) was monitored simultaneously throughout the 30-min hypoxic exerciser period. The test was terminated when one of the symptoms occurred in any participants: performed to volitional exhaustion, ST-segment decreased more than 2 mm, systolic blood pressure > 220 mmHg, or the participant could not keep the exercise load [25].

### 2.5. Evaluation of AMS

Lake Louise scoring (LLS) [26] was used to assess AMS every 1 h during the 6 h of hypoxic exposure (Figure 1A). The positive AMS definition is the four symptoms of headache, nausea/vomiting, fatigue, and dizziness/light-headedness. Participants received a total score ≥ 3 points and headache symptom will be identified as having AMS. Severe headache was also defined as AMS if it obtained 3 points and no other AMS symptoms were present [26]. LLS scale score of 3 to 5 points indicate mild, 6 to 9 points indicate moderate and 10 to 12 points indicate severe AMS.

## 3. Results

At the end of 6-h hypoxic exposure, 7 participants developed AMS (AMS = 7) and 16 participants did not develop AMS (nonAMS = 16). Among the AMS group, we found one particular case patient: the IPAQ-SF showed that the case was physically active (performed high-intensity exercise more than 3 times per week, each session is more than 30 min, the total time of exercise is exceeded 8 h per week) and the interview results revealed that he was a college soccer player, kick in position as a midfield player. However, although this case was physically vigorous, he was an obese patient. His height, weight, waist circumference, body mass index and fat % are 168 cm, 80.2 kg, 87 cm, 28.4 kg/m^2^ and 29%, respectively. In the normoxic base values test, his SpO_2_, HR and BP are 96%, 72 beats per minute (bpm), and 137/76 mmHg, respectively.

At the end of hypoxic exposure, interestingly, physically vigorous did not show a protective effect of AMS. The obese case developed a severe AMS (LLS = 10 points) with symptoms of headache (3 points), cyanosis (1 point), vomiting (1 point), gastrointestinal discomfort (3 points), fatigue/weakness (1 point) and poor appetite (1 point), shown in Figure 1B.

It is worthy of note that, the obese case developed abnormalities of symptoms within the first hour of entering the hypoxic chamber. The case developed severe hypoxemia (SpO_2_ = 70%) with tachycardia symptom (HR = 97 bpm) during the hypoxic rest period. Subsequently developed severe hypoxemia (SpO_2_ = 59%), severe sinus tachycardia (HR = 167 bpm) and BP abnormally elevated (SBP/DBP = 210/91 mmHg) during hypoxic exercise, as shown in Figure 1C–F.

## 4. Discussion

The number of people traveling to high altitudes continues to increase with the growth of tourism and global adventure travel. The participant of high-altitude medicine and physiology is one of increasing importance. As a worldwide epidemic, public health and social problem, obesity plays an important role in the development of high-altitude sickness (acute mountain sickness and chronic mountain sickness). In our study, we reported a physically active obese male suffered from severe hypoxemia and developed severe AMS under hypoxic rest and exercise condition. The case was physically vigorous and usually performed high-intensity exercise more than 3 times per week, with each session lasting more than 30 min and total activity time exceeding 8 h per week. However, our results showed that high physical activity level appears did not protect the obese patient from severe hypoxemia and severe AMS. In view of the physical activity level of our case is likely to be higher than that of typical obese individuals, it is reasonable to speculate that hypoxemia and AMS symptoms are aggravated by hypoxic exposure and hypoxic exercise in most obese individuals.

Hypoxemia is characterized as arterial/peripheral oxygen saturation (SaO_2_/SpO_2_) or arterial partial pressure of oxygen (PaO_2_) level is below the lower limit of normal arterial blood oxygen level. Some observational studies have shown that severe hypoxemia in altitude is a valid predicting tool for AMS in healthy adults [21,22]. However, a recent review has reached an inconsistent conclusion [23], and whether it is applicable to obese people is not clear. We observed SpO_2_ dropped by 26% compared to normoxia (70% vs. 96%) in our case during the 30-min hypoxic rest. Ge et al. [7] showed that obese patients’ nighttime SpO_2_ dropped by 9.7% compared to daytime (76.4% vs. 86.1%). In our case, the obese patient’s SpO_2_ (70%) was obviously lower than AMS patients (means = 75%) and nonAMS patients (means = 77.7%) during the 30-min hypoxic rest. We could infer that obese people are more likely to suffer from hypoxemia. The difference between the obese patient and nonAMS participants was 7.7%, which was slightly higher than that of Ge et al.’s results (6.3%) [7]. It is reasonable to assume that the results of these two studies are relatively consistent, it should be noted that the conditions of the two studies were slightly different (~4400 m of normobaric hypoxia vs. 3568 m of hypobaric hypoxia). Therefore, obese individuals should be cautious to avoid severe hypoxemia in high altitude/hypoxia environments.

Nowadays, although the physiological mechanism is not sufficiently clear, hypoxic therapy, which utilizes hypoxic exposure or hypoxic exercise, or both stimulations combined, is gradually to be widely applied in preventing and treating obesity [9,10,11]. Specifically, this novel regimen has an important clinical utility. However, it should be concerned that hypoxia appears to aggravate obesity-related health risks related to hypoxemia, autonomic nervous system (ANS) dysfunction and AMS symptoms. Based on previous studies and our hypothesis, we observed the obese patient’s SpO_2_ (59%) dropped dramatically by 37% and 13%, compared with normoxia base value (96%) and hypoxia rest (70%), respectively (Figure 1C). And the case developed severe AMS symptoms (LLS = 10). To our limited knowledge, this is the first study on obese patients under dual stimulation with hypoxic exposure and moderate workload exercise (80 watts, 60 rpm). We showed that hypoxemia during hypoxic exercise in obese patients, which may contribute to severe AMS symptoms. The potential reasons might be that the body’s comprehensive compensatory capacity is mobilized for a short period to adapt to homeostasis, and the corresponding indicators become abnormal while the body is unable to achieve homeostasis [5,27,28,29]. A similar result was previously observed in a healthy population by roach et al. [5], but SpO_2_ decreased by only 8%. They believe that hypoxic exercise could better reflect bodies’ hypoxic tolerance. Thus, hypoxic exercise better reflects the relationship between hypoxemia and AMS. Obviously, our result showed that obesity case’s SpO_2_ has a higher drop rate than healthy people. We believe that fat distribution is a potential factor for the higher SpO_2_ drop rate. Previous studies have been reported that the fat mass around the thorax in obese people has a higher correlation with impaired ventilation function and gas exchange ratio [30]. In addition, higher abdominal to waist circumference ratio was negatively associated with forced vital capacity (FVC) and forced expiratory volume in 1 s (FEV1) [31]. We could infer that obesity reduces total respiratory system compliance, which may exacerbate the severity of hypoxemia. Besides this, a pilot study confirmed that, compared with healthy people, obese patients’ chemoceptor sensitivity is higher, and their oxygen dissociation capacity is weaker [7].

Hypoxemia and ANS dysfunction occur first under hypoxia and are considered effective tools for predicting AMS [32,33]. During hypoxia rest, our case developed hypoxemia accompanied by tachycardia (HY-HRrest = 97 bpm vs. NM-HRrest = 72 bpm, Figure 1D). Under the influence of rest or other pathological conditions, hypoxemia can directly lead to compensatory changes in the circulatory system, including the increasing of sympathetic nerve activation, concentration of catecholamines and β-adrenergic receptors in the heart [34,35,36]. It also causes the enhancement of myocardial contractility, HR and venous return [37]. Hypoxia can induce tachycardia by sympathetic activation and vagal withdrawal. Siebenmann et al. [38] found that vagal withdrawal under hypoxic conditions is more likely governed by the arterial chemoreflex activation, rather than a pulmonary inflation reflex in healthy males. Even if sympathetic control is inhibited, hypoxia can still cause tachycardia. Although there is currently a lack of direct evidence, it is reasonable to assume that the above results hold true in the obese population. In addition, tachycardia in our case under hypoxia may be related to the obese people’s poor cardiovascular function [39]. Therefore, ANS dysfunction and the regulation of the autonomic nerve by the vagal under hypoxia are intended to compensate for the exacerbation of hypoxemia.

The results of our case study showed hypoxic exercise not only exacerbates hypoxemia, but also aggravates ANS dysfunction (BPpeak = 210/91 mmHg; HRpeak = 167 bpm, Figure 1D–F). It has been demonstrated that hypoxic exercise exacerbates BP abnormalities in young men [40]. And a prospective cohort study showed that, while hiking (moderate load exercise) at 3700 m altitude, BP abnormalities are higher in overweight and obese people than those in normal-weight individuals [41]. Thus, cardiovascular adaptation to hypoxic exercise is characterized by increased HR and BP due to hypoxia-activated sympathetic nerve [42]. An increase in sympathetic driving leads to a continuously increased BP primarily mediated by cardiovascular contraction and tachycardia [43]. In addition, obese people are more likely to develop metabolic syndrome and BP abnormalities [44,45], which all might be potentially important reasons for abnormal BP while our case performs hypoxic exercise.

## 5. Conclusions

This is the first study to investigate how hypoxic exposure and hypoxic exercise exacerbates hypoxemia and AMS in a physically active obese patient. This case showed that the obese patient is more likely to suffer from severe hypoxemia and ANS dysfunction under hypoxic conditions, and severe AMS symptoms subsequently ensued. The results of the present case study suggest that the physically vigorous does not show a reliable protective effect against severe hypoxemia, ANS dysfunction, and severe AMS symptoms during the 6-h acute hypoxia exposure. Therefore, we conclude that severe hypoxemia is prone to induce AMS in obesity.

In view of severe hypoxemia and ANS dysfunction are observed in the obese patient, who is more prone to develop severe AMS. Our finding provides specific information for the obese people who leisure, trekking, and long-term residence in high-altitude. We recommend that people could conduct an AMS screening procedure before going to high altitude to confirm if they are prone to develop AMS, especially obese individuals. Meanwhile, people who are prone to suffer from AMS should consider taking in some effective pre-acclimatization (such as hypoxic exposure and/or hypoxic training) before going to high altitude. It should be stated that pre-acclimation may not be the optimal choice and weight loss is the preferred regimen. Moreover, hypoxic therapy for the prevention and treatment of obesity needs to be evaluated more carefully. The results in the present case suggest that hypoxic exercise exaggerates severe hypoxemia and AMS symptoms in obese patients, however, the effects of hypoxia dose and training dose remain unclear.

## Data Availability

The data presented in the present study are available on request from the corresponding author.
